# Wild raccoons demonstrate flexibility and individuality in innovative problem-solving

**DOI:** 10.1098/rspb.2024.0911

**Published:** 2024-07-24

**Authors:** Lauren A. Stanton, Carissa Cooley-Ackermann, Emily C. Davis, Rachel E. Fanelli, Sarah Benson-Amram

**Affiliations:** ^1^ Department of Zoology and Physiology, University of Wyoming, Laramie, WY, USA; ^2^ Program in Ecology, University of Wyoming, Laramie, WY, USA; ^3^ Department of Environmental Science, Policy, and Management, University of California Berkeley, 130 Mulford Hall, Berkeley, CA 94720-3114, USA; ^4^ Department of Forest and Conservation Sciences, University of British Columbia, 3041-2424 Main Mall, Vancouver, British Columbia V6T 1Z4, Canada; ^5^ Department of Zoology and Biodiversity Research Center, University of British Columbia, 4200-6270 University Boulevard, Vancouver, BC V6T 1Z4, Canada

**Keywords:** carnivore, cognition, learning, inhibitory control, puzzle box, urban

## Abstract

Cognitive skills, such as innovative problem-solving, are hypothesized to aid animals in urban environments. However, the significance of innovation in wild populations, and its expression across individuals and socio-ecological conditions, is poorly understood. To identify how and when innovation arises in urban-dwelling species, we used advanced technologies and new testing and analytical methods to evaluate innovative problem-solving abilities of wild raccoons (*Procyon lotor*). We deployed multi-compartment puzzle boxes with either one or multiple solution types and identified raccoons using radio frequency identification. Raccoons solved these novel extractive foraging tasks, and their success was influenced by age and exploratory diversity. Successful raccoons always discovered multiple different solution types, highlighting flexible problem-solving. Using a unique, comparative sequence analysis approach, we found that variation in raccoon solving techniques was greater between individuals than within individuals, and this self-similarity intensified during times of competition. Finally, the inclusion of an easier solution in the multi-solution trials enabled previously unsuccessful raccoons to bootstrap their learning and successfully open multiple difficult solutions. Our study suggests that innovative problem-solving is probably influenced by many factors and has provided novel field and analytical methods, as well as new insights on the socio-ecological dynamics of urban populations.

## 1. Background

Urbanization is a dynamic process that results in ecosystem changes and landscape heterogeneity across temporal, spatial and socioeconomic scales [1,2]. The variety of novel conditions and diverse stimuli in urban environments undoubtedly presents wildlife with obstacles, and opportunities, for resource acquisition [3]. Innovation—the creation of a new behaviour or use of an existing behaviour in a new way [4]—is one mechanism by which animals acquire novel or variable resources for survival and may, therefore, be especially relevant in urban environments [5,6]. Indeed, one of the most classic examples of animal innovation, milk bottle opening by songbirds [7], was observed in an urban environment upon the introduction of a novel, anthropogenic resource. Today, humans continue to present urban wildlife with variable opportunities from which foraging innovations arise (e.g. [8,9]); from exclusion structures like chicken coops, to waste receptacles and bird feeders, the diversity of novel and extractable food resources, especially for dietary generalists, seems endless. Such opportunities for innovative problem-solving, alongside other challenges necessitating flexibility in behaviour, have led to the popular notion that urban environments favour species and individuals that are ‘buffered’ by superior cognitive abilities [10,11], perhaps even leading to a cognitive arms race between people and animals vying for resources in cities [12,13]. As such, studying cognitive abilities like innovation in urban wildlife not only allows us to understand the evolutionary effects of urbanization but also empowers us to restore and design cities that meet biodiversity conservation goals and promote coexistence between people and animals [14].

As is the case with other traits, there are costs and benefits to being innovative and thus not all species and individuals share similar levels of innovativeness [15,16]. Across taxa, innovation is correlated with relatively large brains [17–19], which are energetically demanding and result in fitness trade-offs [20,21]. Under the ‘necessity drives innovation’ hypothesis, innovation is expected to arise when established behaviours are unsuccessful [4]. Thus, some individuals that are less competitive at foraging, such as subordinates or juveniles, may have more to gain from innovation (e.g. [22]). Furthermore, innovations involving the incorporation of novel foods or development of novel extractive foraging techniques may be dangerous or too time-consuming for some species and individuals owing to, for example, heightened risks of mortality (e.g. predation and poisoning) or scrounging by competitors [12,23,24]. Even within urban environments, where innovation is predicted to be particularly beneficial [5,10,25–27], we can therefore expect innovativeness to potentially vary across individuals and socio-ecological contexts. However, this possibility has received little empirical attention to date.

In addition, innovations can arise from a process of problem-solving influenced by multiple cognitive and non-cognitive factors [28,29]. Success at innovative problem-solving tasks is typically predicted by a suite of traits including reduced neophobia (avoidance of novelty, [23]), increased exploratory diversity (the total number of unique behaviours exhibited by an individual; also known as behavioural or motor diversity [30]) and persistence (the amount of time spent engaging with a task; also known as work time [28,30,31]). Innovation may also be subject to extraneous, state-dependent sources of motivation, such as hunger (e.g. [32]). For this reason, innovation is often studied in captive conditions, where such extraneous variables may be carefully assessed and/or controlled, using extractive foraging tasks such as puzzle boxes containing a single solution (e.g. [33]). However, captive rates of innovation often surpass what is observed in natural contexts owing to multiple factors like excess energy, time, attention and reduced neophobia, which can lead to biased perspectives of animal cognition in the wild [34]. Furthermore, removing animals from their natural environments for testing dilutes our understanding of how naturally occurring social and ecological conditions may promote, or inhibit, innovation in the wild [24,35,36]. Modern technologies that enable passive (i.e. ‘hands-off’), repeated identification of individuals and can be incorporated into cognitive testing devices, such as radio frequency identification (RFID), have been successfully used with more common study taxa (e.g. birds) but are slow to be extended to less traditional study taxa, such as mammals [36,37]. Thus, our current understanding of the relationship between innovation and urban tolerance could be improved by easing the methodological challenges associated with testing the cognition of free-ranging, urban species, especially those that are more difficult to observe because they are cryptic or nocturnal [36].

Another ecologically relevant aspect of innovation is behavioural flexibility, which is a broad term that refers to an individual’s ability to modify its behaviour in response to change and variation in its environment [38,39]. Individuals in urban environments encounter variability in ecological conditions [25] as well as resources that may require different behaviours and motor patterns for acquisition [12]. If individuals remain solely reliant on previously acquired knowledge and behaviours, then their ability to access novel or changed resources will be limited [11]. Thus, assessing inhibitory control (the ability to resist a prepotent but ineffective response [40]) and repeated innovation (the ability to produce multiple, unique solutions to solve a problem [41]) are important considerations in the study of urban wildlife cognition. A common method of assessing such components underpinning behavioural flexibility is the use of multi-access puzzle boxes that can be solved in multiple ways [42]. This design allows researchers to test whether and how subjects learn multiple solutions to a problem and, therein, demonstrate flexibility in problem-solving. Recently, multi-access puzzle boxes have been successfully used to quantify behavioural flexibility in multiple urban mammalian species including eastern grey squirrels (*Sciurus carolinensis* [43]), brushtail possums (*Trichosurus vulpecula* [44]), spotted hyenas (*Crocuta crocuta* [26]) and captive raccoons (*Procyon lotor* [45]). For instance, when tested with a multi-access puzzle box in captivity, successful raccoons demonstrated behavioural flexibility via repeated innovation, and this was not only predicted by exploratory diversity in the first trial [45] but also correlated with multiple cytoarchitectural characteristics of the brain [46].

Among urban mammals, raccoons stand out for their widespread distribution and local adaptability [47], robust neuronal density [48] and extractive foraging skills [49]. These traits are generally associated with cognitive ability [50,51] and probably help to explain the raccoon’s successful persistence in urban habitats. Indeed, the heightened ability of raccoons to capitalize on abundant, clumped anthropogenic resources likely contributes to the strikingly high densities and aggregations of raccoons in cities [52]. The subsequent interactions occurring among conspecifics and heterospecifics may be altering the sociality and behaviour of raccoons and other urban mesocarnivores in largely unknown ways [53–55], including group foraging and innovation. Only recently, however, have investigations of raccoon cognition in the wild been reported (e.g. [56,57]). One such study illustrated that juvenile raccoons were more willing to make use of a novel food source compared to adults [37], which provides supporting evidence for the necessity drives innovation hypothesis in wild populations. This is a similar finding to a study with captive raccoons, where juveniles were the only individuals that successfully learnt to solve a novel task and demonstrated flexible problem-solving [58]. Thus, raccoons serve as a novel system that can elevate our understanding of intraspecific variation in innovativeness, especially as a function of the unique demands and socio-ecological conditions of urban environments.

Here, we adapted modern technologies and new analytical approaches to assess innovation in a wild population of raccoons using two different puzzle boxes of an original design. These puzzle boxes differ from standard puzzle boxes in that they have 24 locked compartments containing food instead of a single compartment. The first puzzle box presented raccoons with a single-solution type for all compartments, and we predicted that multiple wild raccoons would demonstrate innovative problem-solving by finding the solution. We also predicted that, similar to other studies of innovation, exploratory diversity in the first trial would predict problem-solving success. In accordance with the necessity drives innovation hypothesis, we also expected that juveniles would be most successful at learning to solve the puzzle box and that learning would be demonstrated by a decrease in exploratory diversity and work time across trials. The second puzzle box was similar to a multi-access puzzle box that presented raccoons with four different solution types, with each solution type on 6 of the 24 compartments. We predicted that raccoons would learn multiple solutions, demonstrating flexibility in problem-solving. Because the social environment (e.g. group foraging and competition) can influence the expression of innovation and use of different extractive foraging techniques [9,59–61], we also predicted that the sequence of solution types used would vary within and between individuals and that naturally occurring, competitive testing conditions would influence individual sequences as well.

## 2. Methods

### (a) Study subjects and sites

Our study was conducted in the city of Laramie, Wyoming, during August–October of 2016 and 2017. Prior to the start of testing, we humanely captured (and later released) raccoons using live traps baited with wet cat food to collect demographic information and biological samples. Specifically, we determined the sex (male or female) and age (adult or juvenile) of each individual based on inspection of the genitals, body size, tooth wear and reproductive status (bred versus not bred). We also marked raccoons with passive integrated transponder (PIT) tags via subcutaneous injection between the shoulder blades to facilitate recognition of individuals using RFID techniques. In 2016, we conducted testing at a single location (a residential backyard: *n* = 13 raccoons), and in 2017, we repeated testing at the same location (*n* = 13 raccoons) and added two more locations (a commercial feed store: *n* = 14 raccoons; and an abandoned barn: *n* = 9 raccoons) for a total of three study sites and 39 unique individuals tested. All trapping and experimental methods were approved under the University of Wyoming Institutional Animal Care and Use Committee (most recent protocol no. 20180813 SB00321-02), the Wyoming Game and Fish Department (chapter 33 permit ID: 1019) and are in accordance with the guidelines of the American Society of Mammologists for the use of wild mammals in research [62]. Additional information regarding trapping procedures can be found in Stanton *et al.* [37].

### (b) Procedure

All trials were conducted at night when raccoons are most active. Similar to other recent studies of innovation in the wild (e.g. [26]), we began with a conditioning procedure at each location to attract visitation and encourage participation with the puzzle box (see the electronic supplementary material for conditioning protocol). Each puzzle box was encircled by a flexible antenna (Biomark^®^ Cord Antenna System) attached to a long-range RFID reader (IS1001) that lay flat on the ground to detect the presence of PIT-tagged individuals. We also surrounded the testing area with multiple infrared home security cameras (FLIR FX™ Outdoor Wi-Fi Camera) to record all interactions with the puzzle box (electronic supplementary material, figure S1). We deployed two puzzle boxes approximately 30–40 feet apart at the feedstore and barn to prevent possible monopolization of the box by a single individual, as more space was available at these two locations. We baited puzzle boxes multiple times throughout a testing night and continued nightly testing until all recently detected raccoons had an opportunity to participate or until the threat of inclement weather (e.g. precipitation and cold temperatures) prevented further testing (electronic supplementary material, table S1).

**Figure 1.**
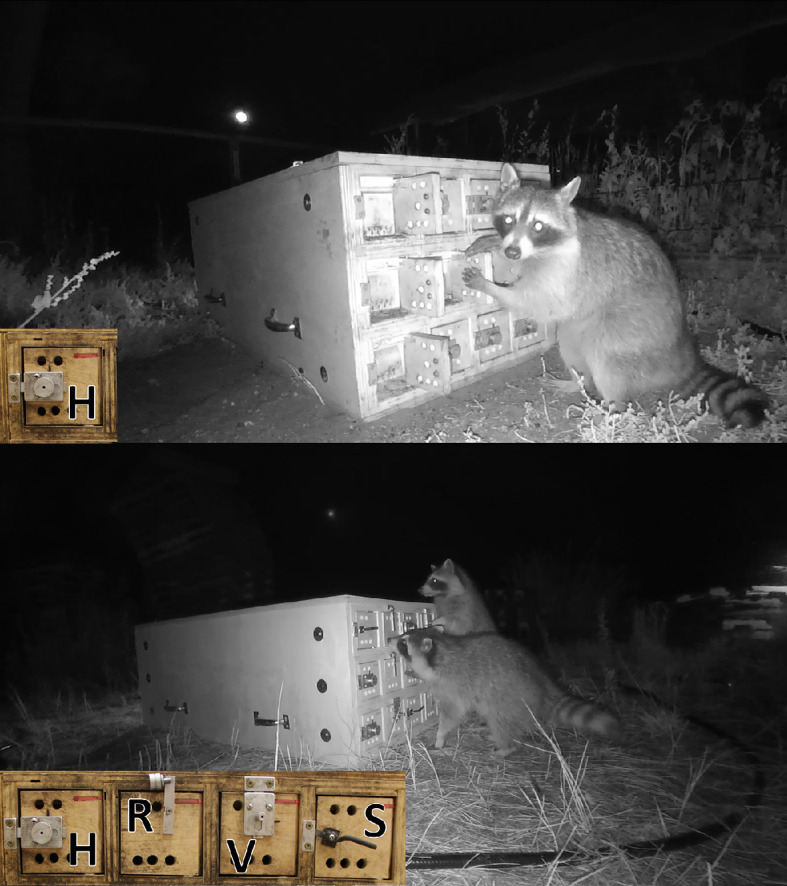
Images of wild raccoons interacting with the single-solution box (top) and the multi-solution box (bottom). All of the doors on the single-solution box could be opened in the same way by sliding the horizontal bolt latch to the right (H). The doors on the multi-solution box could be opened in one of four ways: (H) a side-pull bolt latch (same as the single-solution box), (R) a rod removal and eye hole latch, (V) a pull-down bolt latch, and (S) an up-down swivel latch.

### (c) Single-solution puzzle box

Testing began with a single-solution puzzle box (122 cm × 55 cm × 46 cm) made from plywood that had a series of 12 doors (10 cm × 7.5 cm × 10 cm) arranged in a 3 × 4 grid on the two furthest sides of the box, for a total of 24 doors (figure 1, top). Each door could be opened by sliding the knob of an aluminium bolt latch to the right, revealing a small food reward (i.e. a mix of dog kibble and sardines). This design provided the opportunity for multiple individuals to solve repeatedly within the same night, which allowed us to collect repeated data on wild individuals even if they only participated in testing on a single night. Collecting repeated measures from known individuals was essential, as this allowed us to distinguish learned innovations from accidental openings and one-time discoveries (i.e. improvizations [45]) and examine the process of learning across gained experience [36]. Thus, this novel puzzle box design including multiple doors instead of the standard single door provided wild individuals with multiple opportunities for learning the solution, facilitating greater data collection during a single visit to the box. This novel design also imposed distance between the two working sides of the box, furthering opportunity for multiple individuals to work on the box at the same time, thereby increasing data collection on a larger sample size of individuals.

### (d) Multi-solution puzzle box

After single-solution trials had ended, we introduced the multi-solution puzzle box, which was of similar build and appearance to the single-solution puzzle box but had four different locking mechanisms instead of just one. In the standard multi-access puzzle box paradigm, where a box has multiple doors leading to a single compartment, it is typical to record the number of solutions found overall as an indicator of repeated innovation and to measure the amount of time the animal spends on locked solutions as an indicator of inflexibility and low inhibitory control (e.g. [31,45]). Field testing with wild raccoons did not permit this approach, and we therefore used our multi-compartment design to assess behavioural flexibility by examining the number and preferential order of solutions found. Each of the 24 doors on the multi-solution puzzle box could be opened in one of four ways: (i) a side-pull bolt latch (i.e. ‘horizontal latch’ that was the same as the single-solution puzzle box latch), (ii) a rod removal and eye hole latch (i.e. ‘rod latch’), (iii) a pull-down bolt latch (i.e. ‘vertical latch’), and (iv) a rotating ‘swivel latch’ (figure 1, bottom). Each side of the multi-solution puzzle box contained three horizontal latches, three rod latches, three vertical latches and three swivel latches.

### (e) Scoring and analyses

All statistical analyses were conducted in Program R [63]. A trial was defined as a single visit to the puzzle box, and separate trials were distinguished by the absence of an individual raccoon for least 1 min. Raccoons had to open at least three doors of the same latch type across all of their trials to have demonstrated innovation (i.e. to solve a particular solution and, by extension, solve the puzzle box task). For the single-solution puzzle box, we scored video footage for every identifiable raccoon until they had opened a minimum of 60 doors (see the electronic supplementary material for information on unidentifiable raccoons). We quantified multiple metrics of performance that included (i) the occurrence of door openings during a trial, (ii) exploratory diversity towards the box during a raccoon’s first trial and for every door opened, and (iii) the total amount of time a raccoon spent working to open a door (i.e. work time categorized into four groups: <5, 5–15, 16–30 and >31 s) (electronic supplementary material, table S2). Note that work time was binned and analysed as an ordinal variable, rather than a continuous variable, to achieve the highest inter-observer reliability possible [64].

To evaluate possible predictors of success at solving the single-solution puzzle box, we categorized raccoons as either a solver (1) or non-solver (0) after the completion of single-solution puzzle box testing (i.e. if they opened ≥ 3 doors). We tested whether age (adult and juvenile), sex (male and female), exploratory diversity towards the box in the first trial and first measure of (binned) work time predicted problem-solving success. To do so, we developed and competed binomial generalized linear models (GLMs) using a forward stepwise procedure. We began with an intercept-only model (i.e. null model), and for each step in the model development procedure, we used the MASS package [65] to add the variable that explained the most variation in problem-solving success. We then used Akaike information criterion (AIC) adjusted for small sample size and likelihood ratio tests (LRT) to evaluate relative support for each model [66]. We also tested whether exploratory diversity during the first trial differed between adults and juveniles using a Mann–Whitney *U*-test. Finally, to assess solvers’ learning, we built a cumulative link mixed model (CLMM) [67] and a generalized linear mixed model (GLMM) [68] with work time and exploratory diversity per door as the response variables (respectively) and included door number as a fixed effect with raccoon identity (ID) as a random effect in both models. To avoid any order effects, we limited this analysis to individuals that were first tested with the single-solution box before encountering the multi-solution box. Models were assessed by visual inspection of residuals and additional diagnostic tests where necessary.

For the multi-solution puzzle box, we recorded the sequence of latch types opened by each raccoon during a trial. Because solution difficulty can affect success in puzzle box tasks [45], we first evaluated the difficulty of each latch type by examining the order and frequency of latches opened. We expected that if one or more latches were easier to open in comparison with others, then raccoons would learn to solve these latches first (i.e. opened ≥ 3 doors of the same latch type) and elect to open them first and more frequently overall. We subset the data to include the final five trials for each solver (i.e. a point at which they would have learnt all of the latches that they will ever learn and potentially develop preferences for) when they were the first individual to arrive (i.e. had all doors available to them). We then used *χ*^2^ tests to determine whether the latch frequency differed from than chance for both the first latch opened and the total number of latches opened in each of the final five trials.

Raccoons that learnt more than one latch type across all of their trials were considered to have demonstrated flexible problem-solving. We evaluated behavioural flexibility further by characterizing the extractive foraging techniques used by each individual via a sequence analysis approach, which, to the best of our knowledge, has yet to be applied to puzzle box studies. We calculated the frequency of each latch type opened (i.e. longitudinal entropy) and the number of times raccoons switched between different latches (i.e. transition rate), then combined these measures to calculate a complexity index (0−1) for each trial using the seqici() function in the R package TraMineR [69,70]. When applied within the context of our study, the complexity index reflects an individual’s ability to open and transition between different latches, allowing us to measure flexibility in an extractive foraging technique. A value of 0 represents no complexity, as it indicates that only one latch type was used. By contrast, a value of 1 represents the highest complexity possible and indicates that all latches were used in equal numbers and an individual switched to a different latch every time they opened a door (see the electronic supplementary material for additional details). For this analysis, we only included trials where a raccoon’s testing was unrestricted; that is, where an individual arrived at an unopened box (i.e. all latches available to them), was tested alone (i.e. no other solvers present) and had opened at least six doors to date, as this avoided the inclusion of trials where raccoons had not yet qualified as solvers or instances where a trial was stopped prematurely (e.g. raccoon left the site). To avoid any potential inter-year variation, we limited this analysis to the first year an individual was tested with the multi-solution puzzle box.

We also assessed how similar performance was within and between individuals using an optimal matching edit distance approach [69]. Optimal matching measures the dissimilarity between two sequences in a pairwise fashion, and we used this technique to compare (i) the sequence of latches that an individual opened across its own trials, and (ii) the sequence of latches that an individual opened to that of other individuals across trials. To assess the effects of group foraging, we distinguished between unrestricted trials (i.e. a raccoon was the first solver to arrive and was not joined by another solver during that trial) and trials where competition occurred (i.e. when one or more solvers were present and/or a raccoon was not the first solver to arrive and the box may, therefore, have been partially solved). We then made pairwise comparisons of the sequences within an individual’s own trials (i.e. intra-individual comparisons) and between all of the trials of all individuals (i.e. inter-individual comparisons) for both social conditions (i.e. unrestricted and competitive) and then compared the dissimilarity scores using Mann–Whitney *U*-tests. We performed the pairwise optimal matching distances with the insertion/deletion cost set to 1, and the substitution cost matrix was based on observed transition rates [70]. Like the complexity analysis, we limited this analysis to the first year that an individual experienced both unrestricted and competitive testing conditions, and all sequences were at least six doors long. We also normalized sequence distances to account for varying sequence lengths.

Work time and exploratory diversity for the single-solution trials (*n* = 30 nights of testing) and the sequence of latch types opened for multi-solution trials (*n* = 31 nights of testing) were extracted from video footage by two observers (C.C.-A. and E.C.D.). We calculated inter-observer reliability for three single-solution and two multi-solution nights using correlations for work time (Spearman’s correlation = 0.83), exploratory diversity (Spearman’s correlation = 0.85) and latch type opened (Pearson’s correlation = 0.99).

## 3. Results

### (a) Single-solution puzzle box

We tested a total of 35 raccoons with the single-solution box (electronic supplementary material, video 1), and eight individuals learnt to solve it (23%). For predictors of problem-solving success, our best model included only exploratory diversity (electronic supplementary material, video 2) during the first visit and was superior to the intercept-only (null) model (LRT = 6.55; *p* = 0.01; ΔAIC = 4.6; *w* = 0.81), indicating that raccoons with higher exploratory diversity had a greater chance of success (binomial GLM: *β* = 0.64; confidence interval (CI) = 0.134−1.333; *p* = 0.03; electronic supplementary material, table S3). Exploratory diversity during the first trial was higher in juveniles compared to adults (*W* = 61.5; *p* = 0.04), and, although age did not predict success, a greater proportion of juveniles learnt to solve the single-solution box (15% of adults versus 32% of juveniles). Our assessment of learning showed that both work time (CLMM: *β* = −0.01; CI = −0.013; −0.009; *p* < 0.001) and exploratory diversity (GLMM: *β* = −0.153; CI = −0.207; −0.098; *p* < 0.001) significantly decreased across doors for solvers. One adult female raccoon named Willowherb encountered the single-solution box in both years at the residential backyard (electronic supplementary material, video 3) and her work time on the first door opened was less in 2017 (i.e. < 5 s) compared to 2016 (i.e. > 31 s).

### (b) Multi-solution puzzle box

We tested a total of 31 raccoons with the multi-solution box, and seven raccoons learnt to solve multiple latch types (23%), demonstrating flexibility in problem-solving (electronic supplementary material, video 4). Raccoons typically solved multiple latch types within their first few trials (*x̅* = 3 trials for learning ≥ two latch types). Of these seven raccoons, four had already solved the horizontal latch on the single-solution box, whereas three were novel problem solvers who, despite being present for single-solution trials, had never solved the horizontal latch. All of the successful raccoons solved the swivel, vertical and horizontal latches, whereas only five of the seven raccoons also solved the rod latch. The majority of successful raccoons (*n* = 5) learnt to solve the swivel latch first, even if they already knew the horizontal latch from the single-solution box (electronic supplementary material, figure S2). In the final five trials, the swivel latch was typically opened first (21 initial openings) and in the highest amount (217 total openings), suggesting it was the easiest solution. The swivel latch was followed by the horizontal latch (11 initial openings; 198 total openings), which was known to about half of the solvers, then the vertical latch (three initial openings; 193 total openings). Finally, the rod latch was never selected first (zero initial openings) and was opened less frequently than the other solutions (128 total openings), suggesting it was the most difficult latch. Raccoons did not open each latch at a level expected by chance in their initial opening (*n* = 35; *χ*^2^ = 30.257; *p* < 0.0001) or the total number of openings (*n* = 736; *χ*^2^ = 24.467; *p* < 0.0001).

**Figure 2.**
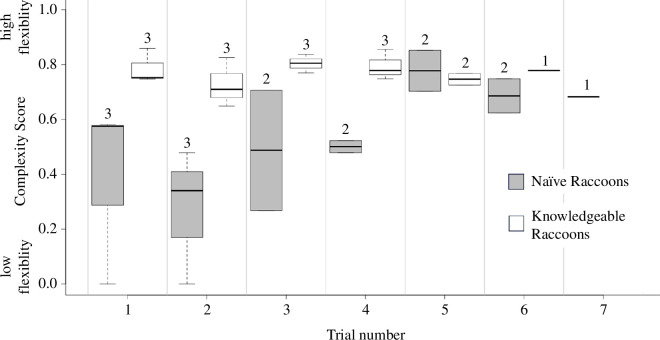
Complexity scores for naïve and knowledgeable raccoons. Raccoons with prior solving knowledge gained from the single-solution box started with higher complexity scores in comparison with naïve raccoons that had no prior experience solving the single-solution box but then increased their scores across trials.

Six raccoons experienced unrestricted testing, and the complexity of their solution sequences varied (median (med) range for all individuals = 0.46–0.84). Knowledgeable raccoons that had learnt the horizontal latch from the single-solution puzzle box started with (med for trials 1–3 = 0.77) and maintained (med for trials 4–6 = 0.77) higher complexity scores across trials, whereas naïve raccoons that had not learnt the horizontal latch started with lower complexity scores (med for trials 1–3 = 0.41) but increased their complexity scores as they gained solving experience across trials (med for trials 4–6 = 0.66) (figure 2).

**Figure 3.**
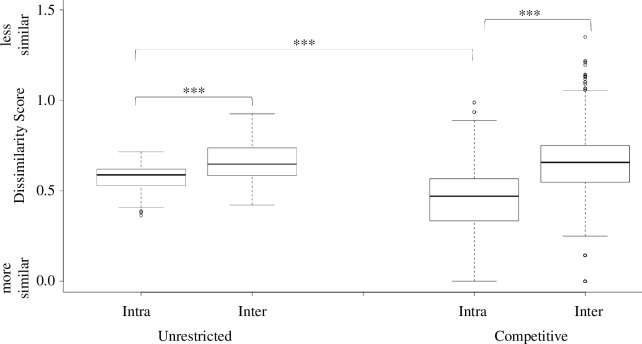
Intra- and inter-individual sequence comparisons across trials in the presence (right) and absence (left) of competition. In both conditions, raccoons showed greater inter-individual variation than intra-individual variation. This difference was more pronounced in competitive conditions, as the intra-individual variation in the presence of competition was significantly less than in the absence of competition (i.e. unrestricted condition; *W* = 128 557; *p* < 0.001).

Raccoons did not open the same order of latches across their own trials, whether unrestricted (med. dissimilarity score = 0.59) or during times of competition (med. dissimilarity score = 0.47) (figure 3). Nevertheless, there was greater dissimilarity between individuals compared to within individuals when raccoons were unrestricted (*W* = 2033.5; *p* < 0.001) and during times of competition (*W* = 1391593; *p* < 0.001). In fact, raccoon performance was more self-similar during times of competition compared to times when they were unrestricted (*W* = 128557; *p* < 0.001; electronic supplementary material, video 5).

## 4. Discussion

In this study, we used novel testing and analytical methods to assess the innovativeness of wild raccoons using puzzle boxes. In line with our predictions, we observed multiple wild raccoons solve the single-solution puzzle box, demonstrating innovation within a novel extractive foraging task. We also found that exploratory diversity best-predicted success and that there was a trend towards greater problem-solving by juvenile raccoons compared to adults. Solvers demonstrated learning of the horizontal latch, as evidenced by a decrease in work time and exploratory diversity across door openings during single-solution puzzle box testing. Furthermore, one raccoon (Willowherb) that was tested with the single-solution puzzle box in both years of the study demonstrated retention of the horizontal latch, indicating that raccoons can remember innovations across long intervals of time. Successful raccoons also learnt to solve multiple latch types on the multi-solution puzzle box, demonstrating flexibility in problem-solving. Finally, sequence analyses revealed that raccoons alternated among latch types in a flexible manner, yet their sequences differed significantly from that of other raccoons, suggesting that extractive foraging can be characterized by both flexibility and individuality.

Our results from the single-solution puzzle box join a growing number of studies that have demonstrated the critical role of exploratory diversity in innovative problem-solving (e.g. [30–32,45,71]). Exploratory diversity may lead to technical innovations that facilitate resource acquisition by urban generalists like raccoons, who forage on diverse food types and extractable sources of human subsidies [12,45]. We also found that juveniles had high exploratory diversity towards the single-solution puzzle box and that a greater proportion of juveniles solved in comparison with adults. This could be because juveniles in this population are in poorer body condition compared to adults and are also more willing to use a novel source of food [37]. Our results may, therefore, offer support for the necessity drives innovation hypothesis [4], in that juvenile raccoons demonstrate greater exploratory diversity and success because they are in greater nutritional deficit. In addition, juvenile animals are often less neophobic than adults, which is attributed to their lack of experience and greater need to gather information about their environment [23,72]. Reduced neophobia is also associated with innovation [28,72]. Although we were unable to measure neophobia directly in this study owing to constraints of the field set-up (i.e. we could not observe when animals first detected the puzzle box, and therefore, the classic measure of latency to approach would not be informative in this study), it is possible that this trait may have also contributed to juvenile interaction and success with the puzzle boxes in our study [24].

Raccoons that successfully solved the multi-solution puzzle box always learnt to open multiple latches, demonstrating flexibility in problem-solving. This result corroborates the previous findings with captive raccoons that have proved capable of learning, and long-term retention of, how to open different types of latches [73] and have demonstrated repeated innovation when tested with a multi-access puzzle box [45]. Interestingly, raccoons did not open the same exact sequence of latches every trial, which we might expect whether they had been following an order of difficulty level or were restricted by a lack of inhibitory control. Although we did find that easier latches were learnt first and preferentially opened more frequently overall, our complexity analysis suggested that raccoons transitioned among the different latch types in a flexible manner. Importantly, raccoons with prior knowledge of the horizontal latch from solving the single-solution box started with high complexity scores at the onset of multi-solution trials, suggesting that they were able to generalize their solving to the new latch types. In this case, it appears that prior knowledge enhanced, rather than inhibited, success at the multi-solution box. This result, along with findings from similar studies (e.g. [31,45]), challenges the role of inhibitory control and behavioural conservatism in repeated innovations [41] or at least within the multi-access puzzle box paradigm. It should be noted, however, that our design incorporated different latches, as opposed to types of openings, which might have permitted similar action patterns and thereby a degree of behavioural conservatism. In contrast to the knowledgeable raccoons, naïve raccoons started with lower complexity scores, learning only one or two latch types initially, but then increased their complexity score as they gained solving experience. Positive feedback gained through experience can indeed promote learning and motivation, which can then lead to innovation [72,74] and greater expertise on a given task [75]. Thus, we believe that the introduction of the swivel latch, which was the easiest of the four latch types, may have afforded a new opportunity for previously unsuccessful raccoons to learn to open doors and provided the motivation to learn additional latch types. This process also illustrates how the initial acquisition of simple associations and skills can quickly lead to greater extractive foraging competency in urban, and other natural, contexts.

Intra- and inter-individual comparisons of sequences revealed that the order of latches opened by an individual across trials was more similar to itself than to other individuals. In other words, the among-individual variation we observed was greater than within-individual variation, suggesting repeatability in performance. Such individual variation in behaviour and cognitive performance is often overlooked, yet is significant because it serves as a pre-requisite for the evolution of cognition [76] and can affect an individual’s fitness (e.g. [16,77]). Of course, it is not currently clear if the observed individual variation in performance in our study, whether it be overall success or preference for different latch types, could yield fitness consequences that selection would act upon. Nevertheless, the patterns we observed may reflect significant, naturally occurring processes in urban systems. For example, individual specialization is one strategy that mitigates intraspecific competition within populations [78] and has important implications for an individual’s cognition. Sea otters (*Enhydra lutris*), for instance, demonstrate extreme individuality in diet and extractive foraging behaviour in response to intraspecific competition [59], including the use of tools when necessary [79]. Similarly, in times of competition, raccoons in our study showed greater individuality in their extractive foraging (i.e. use of latch types) of the multi-solution puzzle box. Although it is currently unknown whether competition in urban environments could be driving individual specialization, evidence of inter-individual variation in mesocarnivore diets suggests that such processes may be occurring (e.g. *Canis latrans* [80] and *Vulpes vulpes* [81]). Thus, our observations of raccoon foraging at the multi-solution box in the presence and absence of competition have revealed exciting insights into group foraging dynamics in urban environments.

Overall, about a quarter of wild raccoons in this study learnt to solve one or both puzzle boxes. This frequency is less than what has been observed in other puzzle box studies with captive raccoons (e.g. 23% versus 65% [45]) probably because the social and ecological dynamics operating in natural, urban environments are absent in captivity. Indeed, other research has demonstrated a positive effect of captivity on innovation [34,82], further illustrating the need to study cognition in a natural setting. For example, scrounging—when an individual exploits the foraging efforts of others [83]—was a frequent occurrence in our study, where an unsuccessful raccoon (i.e. non-solver) gained access to food reward after a successful raccoon (i.e. solver) had opened a door on the puzzle box. Although scrounging can facilitate social learning of a novel foraging technique [84,85], it can also inhibit it [83,86]. Given the communal nature of the food reward in our study (i.e. multiple doors containing multiple pieces of kibble and sardines), it appeared that scrounging provided a successful, alternative foraging strategy that might explain why some individuals never learnt to solve the puzzle boxes themselves. In addition, the costs and perceived risk of interacting with the puzzle box may have created an exploration–avoidance conflict for some individuals, reducing the propensity for innovation [87]. Although urban raccoon survival is probably dependent on their ability to use anthropogenic resources, as a nuisance species, raccoons are also exposed to aversive and lethal stimuli in urban environments, as is the case in our study area (L. A. Stanton, personal observation, 2015-2019). Thus, spending time and incurring (potential) risk by working on the puzzle box may have been perceived as too costly, especially for more experienced adults that probably had alternative, low-risk sources of food available to them.

Despite the benefits of studying cognition in the wild, the inherent lack of control poses a number of challenges and potential sources for bias. For instance, it was impossible to know the background of the raccoons in our study, such as in their rearing or social/lived experience, which could affect performance [88]. Yet, we have taken steps to reduce potential sources of bias in several ways. By working within a single population during a constrained time of year (e.g. non-breeding months in the late summer through to the autumn), we have reduced the potential for variation stemming from seasonal cycles. We also sought to reduce potential biases related to prior experience, order effects and inter-year variation by limiting data contributions to raccoons that met certain criteria in our analyses. Furthermore, other research with this particular population of raccoons did not find an effect of trapping on participation in cognitive testing nor did trapping appear to generate a bias in personality types sampled [37]. In this same study, however, raccoon learning covaried with docility, suggesting a potential link between cognition and emotional reactivity (i.e. ‘coping styles’ [89]). Although it was not possible to evaluate all potential predictors of success in the present study given our relatively small sample size of solvers versus non-solvers, traits related to personality could have influenced our results and would certainly be useful to investigate in the future.

## 5. Conclusion

Despite early and continued observations of innovative foraging in urban environments, the significance of innovation, among other cognitive abilities, for urban species remains unclear [25–27]. Using novel experimental approaches and advanced technologies, we have tested the innovativeness of a wild population of raccoons. In doing so, we have uncovered new insights on the inter- and intra-individual variation in raccoon extractive foraging behaviour. Although innovation is probably influenced by a combination of cognitive and non-cognitive factors, studying the expression of innovation among individuals in natural contexts may more accurately reflect the variation and expression of innovation upon which selection may act [35]. Thus, our study contributes novel techniques and insights on the study of cognition in the wild and highlights exciting future directions on the social and ecological dynamics occurring within urban populations.

## Data Availability

Data and R code are available online [90]. Supplementary material is available online [91].
